# Teratogens: a public health issue – a Brazilian overview

**DOI:** 10.1590/1678-4685-GMB-2016-0179

**Published:** 2017-05-22

**Authors:** Thiago Mazzu-Nascimento, Débora Gusmão Melo, Giorgio Gianini Morbioli, Emanuel Carrilho, Fernanda Sales Luiz Vianna, André Anjos da Silva, Lavinia Schuler-Faccini

**Affiliations:** 1 Instituto de Química de São Carlos, Universidade de São Paulo, São Carlos, SP, Brazil; 2 Instituto Nacional de Ciência e Tecnologia de Bioanalítica, Campinas, SP, Brazil; 3 Departamento de Medicina, Centro de Ciências Biológicas e da Saúde, Universidade Federal de São Carlos, São Carlos, SP, Brazil; 4 School of Chemistry and Biochemistry, Georgia Institute of Technology, Atlanta, GA, USA; 5 Sistema Nacional de Informação sobre Agentes Teratogênicos (SIAT), Hospital de Clínicas de Porto Alegre, Porto Alegre, RS, Brazil; 6 Programa de Pós-Graduação em Genética e Biologia Molecular, Departamento de Genética, Instituto de Biociências, Universidade Federal do Rio Grande do Sul, Porto Alegre, RS, Brazil; 7 UNIVATES University, Lajeado, RS, Brazil

**Keywords:** Birth defects, teratogens, Zika virus, pregnancy, public health

## Abstract

Congenital anomalies are already the second cause of infant mortality in Brazil, as in many other middle-income countries in Latin America. Birth defects are a result of both genetic and environmental factors, but a multifactorial etiology has been more frequently observed. Here, we address the environmental causes of birth defects – or teratogens – as a public health issue and present their mechanisms of action, categories and their respective maternal-fetal deleterious effects. We also present a survey from 2008 to 2013 of Brazilian cases involving congenital anomalies (annual average of 20,205), fetal deaths (annual average of 1,530), infant hospitalizations (annual average of 82,452), number of deaths of hospitalized infants (annual average of 2,175), and the average cost of hospitalizations (annual cost of $7,758). Moreover, we report on Brazilian cases of teratogenesis due to the recent Zika virus infection, and to the use of misoprostol, thalidomide, alcohol and illicit drugs. Special attention has been given to the Zika virus infection, now proven to be responsible for the microcephaly outbreak in Brazil, with 8,039 cases under investigation (from October 2015 to June 2016). From those cases, 1,616 were confirmed and 324 deaths occurred due to microcephaly complications or alterations on the central nervous system. Congenital anomalies impact life quality and raise costs in specialized care, justifying the classification of teratogens as a public health issue.

## Background

Congenital anomalies are among the major causes of infant death worldwide (Brazilian Ministry of Health, MoH) (Egbe *et al.*, 2015). In the United States, as in other high-income countries, birth defects are the main cause of infant mortality, being responsible for 1 out of 5 infant deaths (Petrini *et al.*, 2002). Within the main causes of infant deaths in the United States, congenital malformations, deformations and chromosomal abnormalities appear in first place, representing 20.3% of total infant deaths in 2013 (Xu *et al.*, 2016). These are also the reasons for 12% of all pediatric hospitalizations (Egbe *et al.*, 2015). In Latin America, congenital anomalies are between the second and fifth cause of death in children under 1 year of age (Bronberg *et al.*, 2014). In Brazil, since 2000, they are the second main cause of infant death (Passos-Bueno *et al.*, 2014), and among the three leading causes of infant hospitalizations, responsible for 37% of pediatric hospital admissions. In addition, the hospital mortality rate in children with malformations accounted for 9.8% of total deaths, almost twice as in children without malformations (Horovitz *et al.*, 2005).

Congenital anomalies affect about 1 in 33 liveborns, with an estimated 3.2 million newborns with birth defects per year. Moreover, in 2013 a worldwide estimate showed that nearly 276,000 newborns die before one month of life every year, as a result of these congenital anomalies (World Health Organization, 2015a). From those, 10 to 25% present specific genetic inheritance, 10% are caused by environmental factors, such as teratogens exposure, and 65 to 75% remain with unknown causes, what may include polygenic diseases, multifactorial factors (gene-environment interactions), spontaneous development abnormalities and synergistic interactions with teratogens (Brent, 2001).

Teratogens are environmental agents such as drugs, viruses, lack of nutrients, and physical or chemical elements that upon contact with embryo/fetus can cause congenital anomalies, generating permanent functional or morphological changes in the newborn (Shepard, 1982).

Among the main reasons for pregnant women having contact with teratogenic substances is the association of preexisting public health problems (such as lack of medical care, drug and alcohol consumption, lack of basic sanitation) to other social issues like poverty and illiteracy (Reidpath and Allotey, 2003; World Health Organization, 2015a). Therefore, the offspring of socially disadvantaged women are more vulnerable to birth defects, causing an impact on infant mortality and health expenses with specialized care, qualifying teratogens as a public health problem.

## Teratogenic agents

Ancient Egypt wall paintings suggest the long time existence of congenital abnormalities, such as clubfoot and achondroplasia (Barrow, 1971). Children with congenital malformations were labeled as “monsters” by the ancients (Garcias and Schüler-Faccini, 2004). Other regions, such as the Americas, Australia and Pacific Islands also have early records of primitive sculptures revealing concerns with congenital anomalies, which have even inspired some mythological figures (Barrow, 1971). Only in the 1930s malformations started to be scientifically studied in animal models. Pioneer studies were performed in pig offspring, addressing dietary vitamin A deficiency in pregnant sows, which caused a complete absence of eye globe and ocular tissue in the newborns (Hale, 1933).

Between the 1950s and 1960s thalidomide was extensively used as sedative and to treat morning nausea during pregnancy in Europe, Australia, Canada, Japan and Brazil. Thalidomide use during pregnancy caused limb reduction defects in thousands of newborns, leading to its ban in most countries since 1961 (Kim and Scialli, 2011). The thalidomide tragedy caused an increasing interest about drug exposure during pregnancy and the mechanism of action of teratogenic agents on embryo-fetal abnormality development.

Humans are exposed to millions of potential deleterious substances and hazardous conditions daily. However, only a small part of these substances have been tested in animals and even fewer were confirmed as a teratogenic for humans, as teratogenicity studies cannot be conducted in humans due to ethical reasons (Kalter, 2003).

## Principles of Teratology

Teratology studies establish relationships between environmental agents and anatomical and physiological changes in the fetus (Finnell, 1999; Kalter, 2003). We present below the six basic principles that determine teratogenic effects (Wilson, 1977; Finnell, 1999).

### Maternal-fetal genotype

A teratogenic effect depends on maternal-fetal genotype and on how embryos interact with their surroundings. Due to these conditions, newborns exposed to a specific dose of the same teratogenic can show different phenotypes. Teratogens are capable of interacting with some genes, modifying morpho-functional patterns and resulting in either a major susceptibility or resistance to harmful substances. Some biochemical pathways can also respond in distinct ways to different agents, affecting even more malformation patterns (Cassina *et al.*, 2012).

### Mechanisms of action

The most common mechanisms of action of teratogens are hyperacetylation, cholesterol imbalance, alteration of folate metabolism and folate antagonism, retinoic acid imbalance, endocrine disruption, vascular disruption and oxidative stress (Giavini and Menegola, 2012).

#### Hyperacetylation

Hyperacetylation may occur due to the inhibition of histone deacetylase enzyme (HDAC), and the acetylation status of the histone affects the modulation of chromatin structure and gene expression, interfering with the embryonic development. HDAC inhibitors have been used as anticonvulsant drugs (valproic acid) and for cancer treatment, once they prevent tumorigenesis. Among anticancerdrugs are trichostatin A (TSA), apicidin and sodium butyrate. These drugs are able to induce hyperacetylation in animal embryos, leading to congenital malformations, such as neural tube and axial skeletal defects (Menegola *et al.*, 2005).

#### Cholesterol imbalance

A high amount of cholesterol is required for fetal development. This biomolecule is supplied by the mother during early pregnancy and transported to the fetus across the placenta, while during late pregnancy, cholesterol biosynthesis will depend on the fetus’ own production (Waterham, 2006). Drugs used for the treatment of hypercholesterolemia, such as statins, act by blocking HMG-CoA reductase, the enzyme involved with cholesterol biosynthesis. HMG-CoA reductase is converted to mevalonate (Charlton-Menys and Durrington, 2008), interrupting the synthesis of cholesterol, and thus can cause adverse effects on the developing fetus (Edison and Muenke 2004).

#### Alteration in folate metabolism and folate antagonism

Folate or water-soluble vitamin B acts as a co-enzyme in biochemical reactions, as a receiver or donor of one-carbon units, and it is involved in purine and pyrimidine synthesis and in DNA methylation. Increased cell growth and tissue proliferation during embryogenesis demands an increase in DNA synthesis, for which the presence of folate is essential. Some drugs can compete with dihydrofolate reductase and block the conversion of folate to tetrahydrofolate. Among these drugs are methotrexate, sulfasalazine, triamterene and trimethoprim. Other drugs, such as anti-epileptic drugs, can interfere in folate absorption or influence folate degradation (including valproic acid and carbamazepine phenytoin). The most common birth defects involving these drugs are neural tube defects, orofacial clefts and limb defects (van Gelder *et al.*, 2010).

#### Retinoic acid imbalance

An imbalance between synthesis and degradation of retinoic acid can lead to an excess or deficiency of this acid, resulting in deleterious effects on cells and embryos, once this Vitamin A precursor is closely related to vertebrate morphogenesis. However, retinoic acid is also a signaling molecule in neural crest cells, which will originate various cell types and structures, such as intramembranous bone, cartilage, peripheral nerves and Schwann cells, and muscles, amongst others. Drugs such as isotretinoin can contribute to retinoic acid imbalance and lead to craniofacial and axial skeleton malformations (Giavini and Menegola, 2012).

#### Endocrine disruptors

Endocrine disruptors may interfere with the release of hormones and in reactions mediated by hormone receptors (van Gelder *et al.*, 2010). Diethylstilbestrol, oral contraceptives, fertility treatment drugs and other endocrine disruptive chemicals, which can include bisphenol A and phthalates, act as endocrine disruptors (van Gelder *et al.*, 2010). These teratogenic substances can cross the placenta and lead to fetal genital malformations (Raman-Wilms *et al.*, 1995)

#### Vascular disruption

Changes in the development of veins, arteries and capillaries will disturb blood perfusion in fetal tissues. These maternal-fetal blood disturbances can include hyperperfusion, hypoperfusion, hypoxia and obstruction, and are caused by anatomical problems, maternal chronic diseases or exposure to teratogenic agents during pregnancy, such as misoprostol, phenytoin, cocaine, ergotamine, and some vasodilator and vasoconstrictor drugs. Structural birth defects are the most commonly reported, particularly limb defects (Holmes, 2002).

#### Oxidative stress

**Figure 1:**
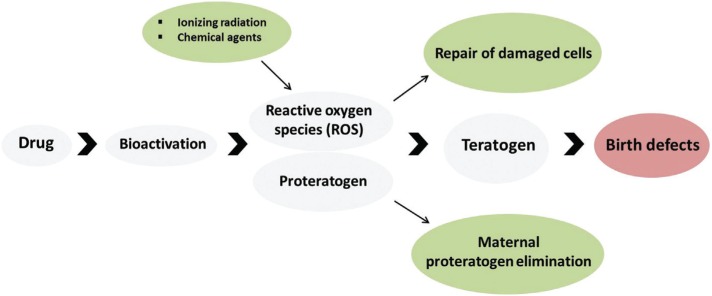
Teratogenesis pathways due to oxidative stress.

Oxidative damage to cellular macromolecules such as lipids, proteins, DNA and RNA is caused by reactive oxygen species (ROS), which provide oxidation-reduction reactions (Wells *et al.*, 1997). Exogenous ROS sources include ultraviolet light (UV), UVA and UVB radiation, ionizing radiation, and chemical agents, while endogenous sources are related to cellular metabolism or oxidase enzymes (Hansen, 2006; van Gelder *et al.*, 2010; Giavini and Menegola 2012). Some of these agents (also called proteratogens) can be bioactivated by embryonic cytochrome P450 enzymes. Their teratogenic effect will depend on the intracellular balance between proteratogen bioactivation, molecular target damage, maternal proteratogen elimination, and repair of damaged cells (Winn and Wells, 1995; Wells *et al.*, 1997), as illustrated in Figure 1. Among drugs that induce oxidative stress are thalidomide, valproic acid, phenytoin, alcohol, (van Gelder *et al.*, 2010), and anticancer drugs (Conklin 2004).

### Conceptus development stage

**Figure 2:**
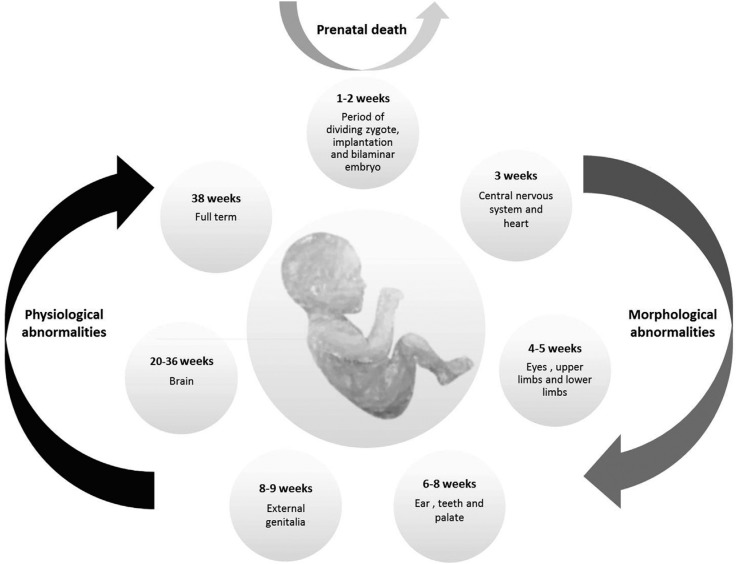
Critical stages of human embryological development.

Organisms present distinct sensitivity to external agents according to their gestational age. A conceptus is a fertilized egg cell until the 3^rd^ week of gestation. The period from the 3^rd^ to the 8^th^ week it is called embryonic phase, and from the 9^th^ week onward the fetal phase (Niakan *et al.*, 2012). The critical phases of gestation are represented in Figure 2.

The two initial weeks after fertilization, in which the zygote is undergoing mitotic cell division is called the ‘all-or-nothing’ phase; in case a contact with a teratogenic agent occurs, it can result either in spontaneous abortion or in a normal embryo-fetal development. If teratogenic exposure occurs between the 3^rd^ and 8^th^ week of gestation, a period in which most of the morphological structures develop, it can lead to considerable phenotypical changes in the embryo, such as alterations in the central nervous system, limbs and face. From the 9^th^ week of gestation some organs are still developing, like external genitalia and brain, and exposure to teratogens can culminate in functional abnormalities. However most morphological characteristics are preserved from this phase onward (Shepard, 1979; Niakan *et al.*, 2012).

### Nature of the agent

Teratogenic agents can affect the embryo in different ways, according to the teratogen's nature. Physical teratogenic agents, such as ionizing radiation, affect the embryo directly. Drugs and other chemical substances on the other hand are previously metabolized by the mother's organism before reaching the fetus. This metabolism can either activate or inactivate relevant metabolites, resulting in different teratogenic susceptibilities (Finnell, 1999; Gilbert-Barness, 2010; Niakan *et al.*, 2012).

### Dose-response relationship

Considering basic principles of dose-response, prolonged teratogen exposure leads to worsened embryo-fetal sequelae. Dose is also a major factor, with evidence of short exposures to high teratogen doses leading to more deleterious effects (Cohlan, 1963; van Gelder *et al.*, 2010).

### Final manifestation

The resulting teratogenic effects are spontaneous abortion, fetal loss, embryo-fetal morphological abnormalities, intrauterine growth restriction, and functional disabilities, such as intellectual disability (Finnell, 1999; Gilbert-Barness, 2010).

Teratologists use Shepard's seven criteria to establish human teratogenicity (Shepard, 1994): 1) proven exposure to the agent during the critical stages of prenatal development; 2) consistent findings on two or more high-quality epidemiological studies (control of confounding factors, sufficient number of cases, exclusion of positive and negative bias, prospective studies, if possible); 3) careful delineation of clinical cases; 4) rare environmental exposure that is associated with the rare defect (three or more cases, at least); 5) teratogenicity in experimental animals; 6) the association should make biological sense; and 7) experimental proof that the agent acts in an unaltered state. Criteria 1-4 are considered essential. criteria 5, 6, and 7 are helpful but not essential.

## Teratogen categories

Teratogenic agents can be categorized into: I) drugs and substances, II) physical agents, III) environmental agents, IV) maternal infections, and V) maternal conditions (Gilbert-Barness, 2010). Table S1 (supplementary material) presents 44 teratogenic agents and their characteristics, as well as their respective birth defects.

## Teratogenic risks

Due to concerns about teratogenic risks regarding medicinal drugs, the Food and Drug Administration (FDA) created a risk classification for these substances during an international symposium of the Teratology Society in 1992 (Alván *et al.*, 1995). Substances are classified following a progressive risk order (A, B, C, D and X) (Food and Drug Administration, 2014a; Alván *et al.*, 1995): A) controlled studies did not show risk to the fetus; B) absence of risk to humans or results from tests on animals did not show fetal risk; C) risks cannot be rejected; controlled studies in humans are scarce or inexistent; although animals studies had positive results, there is no human data available; there may be adverse effects on the fetus (drug benefits must justify fetal risk; D) studies have shown risk to the fetus (substance prescription depends on the mother's need, to preserve the mother's life); X) contraindicated in pregnancy; risk to the fetus is greater than the benefit of the drug.

However, as this classification allowed for misinterpretation and errors in prescribing decisions, the FDA removed pregnancy letter categories (A, B, C, D, and X), and published a new final rule, the “Pregnancy and Lactation Labeling Rule” (PLLR), for classification based on a narrative structure rather than a category system, which provides a clearer description of potential risks of drug exposure during pregnancy. From June 30, 2015, these labeling changes came into effect, to which prescription of drugs and biological products have to comply. This final rule requires the use of the following subsections: I) Pregnancy - information about use of the drug in pregnant women, which includes the dosing and potential risks to the developing fetus; information about registries of pregnant women affected by a drug or biological product, and a recommendation of inclusion in the drug label of the existence of any pregnancy registries; II) Lactation - information about using the drug while breastfeeding, which includes the amount of drug in breast milk and possible effects on the breastfed child; III) Reproductive Potential of Females and Males - information about the need for pregnancy testing, contraception recommendations, and infertility related to the drug (Food and Drug Administration, 2014b).

## Why are teratogens a public health problem?

Preexisting public health problems, such as alcohol and drug consumption, malnutrition of pregnant woman (Scholl and Johnson, 2000), precarious health conditions, lack of infrastructure and information (McMichael *et al.*, 2005) contribute to the contact of pregnant women with teratogenic agents and may interact as risk factors for fetal outcome. Other factors, such as illiteracy, familiar issues and low income aggravate the situation (Nutbeam, 2006), impacting infant mortality and costs on specialized medical treatment, and aggravating other public health issues.

## Survey of birth defects cases in Brazil

The survey data are from the online platform of the Brazilian Ministry of Health (MoH) based on records of the Unified Health System (SUS). DATASUS (http://www2.datasus.gov.br/DATASUS/index.php?area = 02) is a computerized system that provides a database with information about birth statistics (mortality and live births), epidemiology and morbidity, health indicators, and demographic and socioeconomic information.

**Figure 3:**
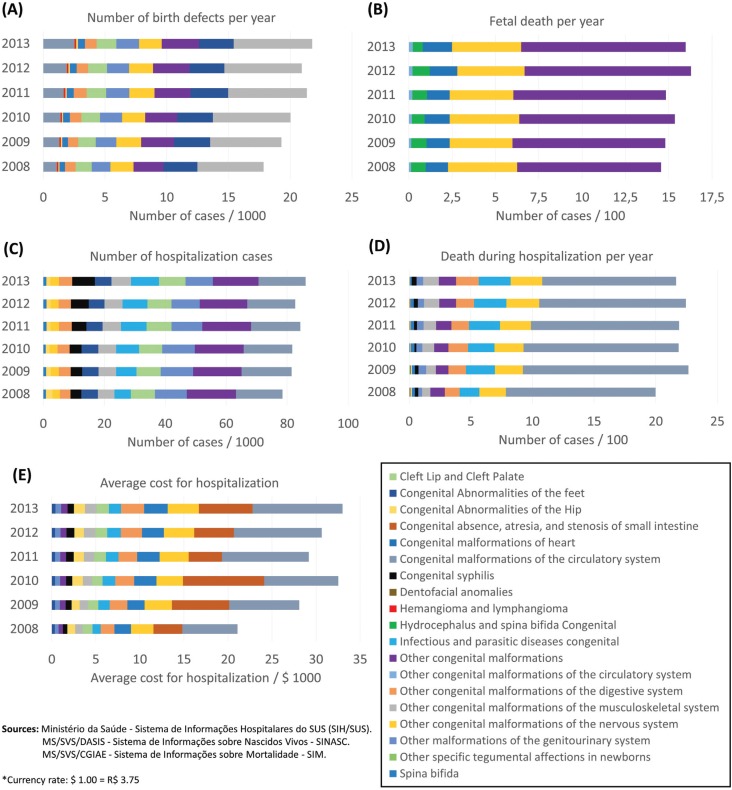
Comprehensive survey of birth defects in Brazil from 2008 to 2013. A) Number of children born with birth defects. B) Number of fetal deaths. C) Number of hospitalizations involving birth defects per year. D) Number of deaths of hospitalized patients due to birth defects. E) Costs associated with treatment and on hospitalization's admissions.

The SINASC (Information System of Live Births) and SIM (Mortality Information System) databases, which have information about the number of cases of children born with birth defects (Figure 3A) and the number of fetal deaths (Figure 3B) from 2008 to 2013, registered 121,233 infants with congenital anomalies, an annual average of 20,205 cases (Figure 3A). If we consider that the Brazilian annual birth rate average of this period is about 2,900,000 (SINASC), there is a 0.7% prevalence of births with anomalies. Although this may seem a small number, it is worth noting that the ~20,000 infants born with anomalies every year directly impact the public health system. However, studies have shown that there is a clear underreporting, as the rate for birth defects in humans is around 3% (Parker *et al.*, 2010; Xu *et al.*, 2016). Depending on the disability, these children will require specific treatments, ranging from educational specialists, to physical therapy, or surgical interventions. Moreover, in addition to these birth defects there were 9,178 fetal deaths due to congenital anomalies reported during the same period, corresponding to an annual average of 1,530 (Figure 3B).

Another survey was performed in the SIH/SUS (Hospital Information System/ Unified Health System) databases, using as a parameter the hospital morbidity lists by ICD-10 (International Classification of Diseases) from 2008 to 2013. The following variables were searched: infant hospitalizations involving birth defects per year (Figure 3C), deaths of hospitalized patients due to birth defects (Figure 3D), and average cost of hospitalizations due to birth defects (Figure 3E). Figure 3C shows that during this period there were a total of 494,714 infant hospitalizations due to birth defects, which represents an annual average of 82,452.

There were 13,050 reported deaths (Figure 3D), with an average of 2,175 per year. Thus, the number of children dying after hospitalization is higher than the number of fetal deaths due to congenital anomalies. About 3% of children who were hospitalized with birth defects died, which, added to the 1,530 fetal deaths, totals 3,705 annual deaths. The total cost associated with infant hospitalization involving congenital anomalies was 46,550 USD during these six years (Figure 3E), corresponding to an annual average of 7,758 USD.

Furthermore, in addition to hospital costs, patients with chronic conditions will need continuous treatment, increasing even more the associated costs. These costs can include drugs, physiotherapy, speech therapy, occupational therapy and special education, and in many instances the mother quits her job to care for the child (Horovitz *et al.*, 2005).

Tables S2-S6 are attached as supplementary material and contain the raw data (2008 to 2013) from the Brazilian Ministry of Health website. As can be seen, there is a lack of specification in the records jeopardizing the identification of the etiology of the possible teratogens. Better epidemiological information would be very important for the detection of new teratogens, as well as for their proper control. The microcephaly epidemics, as we will show below, is an example of how the lack of proper registries in many countries made it difficult to identify the real impact of the Zika virus infection in pregnancy and the extent of the resulting birth defects.

## Brazilian cases of teratogenesis

### Microcephaly outbreak in Brazil and Zika prenatal virus infection

In 2015 there was a sudden appearance of Zika virus (ZIKV) infections in Brazil, especially in the Northeast region (Campos *et al.*, 2015). Climatic factors, lack of infrastructure, and the population's negligence offered favorable conditions for the proliferation of *Aedes sp.* mosquitoes, the Zika virus vector (Costa *et al.*, 2008). Furthermore, recent studies suggest a potential sexual transmission of Zika virus (Musso *et al.*, 2015; Atkinson *et al.*, 2016; D’Ortenzio *et al.*, 2016), increasing the concern received from public authorities. The principal reason for alarm is the increased risk of the unborn babies to develop neurological and brain abnormalities, characterized by microcephaly, when infected by Zika virus during pregnancy (Mlakar *et al.*, 2016; Rasmussen *et al.*, 2016).

From 2010 to 2014 there was an average of 160 cases of microcephaly per year in Brazil (Brazil Ministry of Health, 2015). A drastic increase was reported from October 22, 2015 to June 18, 2016, with 8,039 suspected and 1,616 confirmed cases of microcephaly (Brazil Ministry of Health, 2016). The cases occurred in 576 municipalities, throughout all 26 Brazilian States and the Federal District. In a study analyzing the first 1,501 cases of microcephaly reported to the Brazilian MoH, 602 were included as possibly related to ZIKV prenatal infection (depending on the level of laboratorial, radiological or clinical evidence) (França *et al.*, 2016).

Microcephaly can be caused by genetic, environmental or multifactorial causes (Alcantara and O’Driscoll, 2014). The astonishing increase in the reported number of cases of microcephaly in 2015 led the Brazilian MoH to declare state of emergency in the country (Brazil MoH, 2015), and on February, 2016, the World Health Organization (WHO), in turn, declared the large number of cases of microcephaly and neurological disorders and their possible relationship with ZIKV a Public Health Emergency of International Concern (PHEIC) (World Health Organization, 2016).

Soon after, the ZIKV genome was detected in the amniotic fluid of pregnant women whose fetuses presented microcephaly (Calvet *et al.*, 2016), as well as in the brain of aborted fetuses, in urine, and in cerebrospinal fluid (Brasil *et al.*, 2016; Mlakar *et al.*, 2016).

Rasmussen *et al.* (2016), using Shepard's criteria for defining a human teratogen, concluded that there was enough evidence to establish the causal relationship between ZIKV infection during pregnancy and microcephaly, in addition to other brain anomalies. Studies in mice revealed that the Brazilian Zika virus (ZIKVBR) is capable of infecting the fetus, leading to intrauterine growth restriction, including microcephaly (Cugola *et al.*, 2016). Other studies showed that ZIKV targets human brain cells (Garcez *et al.*, 2016), clearly revealing the association between ZIKV infection during the first trimester of pregnancy and microcephaly risk (Johansson *et al.*, 2016).

Microcephaly can lead to several complications, such as intellectual disability, growth retardation, strabism, epilepsy, metabolic disorders, and cerebral palsy (Watemberg *et al.*, 2002; Ashwal *et al.*, 2009), with an enormous economic and social impact for the country in future years. Therefore, effective actions should be implemented immediately to contain the ZIKV spread. Those actions should include better access to public sanitation and health policies, control of the *Aedes* vector, which also transmits other tropical diseases such as dengue and yellow fever, along with the search for effective vaccination and pharmacological treatment.

### Misoprostol

In developing countries there is an early sexual initiation and lack of family planning. Women tend to be aware of pregnancy weeks or even months later (De Moura and Gomes, 2014), and there is a high rate of undesired pregnancies.

In countries were abortion is illegal, as in Brazil, intentionally induced clandestine abortions contributes to high numbers of maternal mortality. Around 5% of Brazilian women end up opting for the illegal use of misoprostol to terminate unwanted pregnancies (Vargas *et al.*, 2000). This medical drug is a synthetic prostaglandin E1 analog, originally prescribed to treat gastric ulcers, which stimulate uterine contractions, leading to miscarriage. In Brazil, it has been banned from the market because of its use as an illegal abortion method (Costa, 1998; Schuler *et al.*, 1999; da Silva Dal Pizzol *et al.*, 2006; Allen and O’Brien, 2009). Misoprostol alone does not present great efficiency in inducing abortions, and can induce fetal abnormalities, especially if used in the first trimester of pregnancy (Gonzalez *et al.*, 1998; Costa, 1998; da Silva Dal Pizzol *et al.*, 2006; Allen and O’Brien, 2009). Furthermore, the increased use of drugs or herbs with perceived abortive actions by the population highlights the lack of control in selling and using prescription medications.

A cohort study of pregnant women treated in prenatal services in six Brazilian capitals revealed that 707 women used products to induce menstruation, which includes herbal teas (34.4%), sex hormones (28.3%), and misoprostol (17%) (Pizzol *et al.*, 2008). The congenital anomaly risk was 2.74 times greater for fetuses exposed to misoprostol when compared to unexposed fetuses. Misoprostol is associated mainly to congenital paralysis of the 6^th^ and 7^th^ cranial nerves and to limb reduction defects due to fetal vascular disruption (Gonzalez *et al.*, 1998; Vargas *et al.*, 2000).

### Thalidomide

Even after the immense international repercussion of the teratogenic potential effects of thalidomide, many cases of newborn malformation involving this medicine were registered in Brazil in different years. Thalidomide is a drug indicated for the treatment of erythema nodosum leprosum (ENL), and more recently, to a number of different medical conditions, due to its immunomodulatory properties (Vianna *et al.*, 2011, 2015; Kim and Scialli, 2011;). The Latin-American Collaborative Study of Congenital Malformations (ECLAMC) reported 34 thalidomide embryopathy cases in South America from the 1960s to 1990s, of which 33 where in Brazil. Most of these cases involved mothers treated for leprosy, whose babies presented birth defects, such as phocomelia, hypoplastic glenoid, absence of thumbs, absence or hypoplasia of radius, a third arm bone, and polydactyly (Castilla *et al.*, 1996).

Three cases were reported from 2005 to 2010. From those, four involved mothers that underwent treatment for ENL and were unaware of pregnancy, while the third case involved self-medication of a pregnant woman who took thalidomide prescribed for her mother, who was being treated for multiple myeloma (Schuler-Faccini *et al.*, 2007). The majority of cases involves lack of medical information, which is related to public health.

Despite safety concerns, the Brazilian population has a high consumption of thalidomide. The state of São Paulo leads the thalidomide drug distribution rates (5,889,210 thalidomide tablets), followed by Minas Gerais and Rio de Janeiro, from 2005 to 2010. In this period, there were 2,802 reported cases of limb reduction defects, with 192 cases compatible with a thalidomide embryopathy phenotype (TEP) (Vianna *et al.*, 2015). This demonstrates the seriousness of this public health issue.

### Alcohol

In 2012, alcohol was responsible for almost 3.3 million of deaths, corresponding to 5.9% of the total number of deaths worldwide. Excessive alcohol consumption prevails among adults aged 20-39 years, although alcohol use by young people starts at early ages – even from 12.5 years old (Vieira *et al.*, 2007; World Health Organization, 2015b). Alcohol abuse during pregnancy can lead to Neonatal Abstinence Syndrome in babies. Alcohol is the teratogenic agent responsible for the Fetal Alcohol Syndrome (FAS), as well as Fetal Alcohol Spectrum Disorders (FASD), being a major non-genetic cause of intellectual disability and behavioral problems (Abel and Sokol, 1987; Momino *et al.*, 2012; Chapman and Wu, 2013; O’Leary *et al.*, 2013).

In 2015 a study was carried out in a Brazilian orphanage in Recife to investigate the frequency of FASD. Children were evaluated by a multidisciplinary team, and the following results were obtained: 50% of the childrens mothers were reported as known alcohol abusers. Of these children, 18% presented general developmental delay, 3% had intellectual disabilities, 27% had cognitive impairment, 14% had attention deficit/hyperactivity, and 3% presented autism. A total of 17% presented FASD, three children presented FAS, six presented partial FAS, and seven presented neurological disorder related to alcohol. About 16% of these children presented ocular changes, such as low vision, strabism and morphological changes of optic nerves, which shows the devastating effects of drug abuse during pregnancy. (Strömland *et al.*, 2014)

### Illicit drugs

Illicit drug consumption during pregnancy is another public health problem involving a potential embryo-fetal effect. The effects of these substances include low birth weight, intrauterine growth restriction and placental abruption, as well as premature birth or spontaneous abortion (Holbrook and Rayburn, 2014). Cocaine and its derivatives, such as crack and heroin, may have harmful effects on pregnancy (Cherukuri *et al.*, 1988; Rizk *et al.*, 1996; Costa *et al.*, 2012). The extent of the problem can be estimated by the increase in the demand of medical care and costs with hospitalization and specialized treatment for drug-addicted pregnant women, as well as the increase in the number of premature births. Another issue is the increased rate of sexually transmitted diseases or other maternal infections related to illicit drug and alcohol use (Tüzün *et al.*, 1999; Hwang *et al.*, 2000), which can also be teratogenic, like syphilis.

## Concluding remarks

This review presented fundamental aspects of teratogenic agents, bringing an overview about the number of cases of congenital anomalies, and their contribution to an increase in mortality rates, hospitalizations and treatments expenses for the Brazilian Unified Health System. We also present cases of congenital malformations involving teratogens, as well as the definitive classification of Zika virus infection as a teratogen and now a real public health problem.

Congenital anomalies caused by teratogenic agents are essentially avoidable, with a great impact on public health, on the economic and on social aspects. Public policies to prevent, care for, and treat these disabilities are extremely important to manage this public health issue. To address these problems, a collaborative agreement among the United Nations (UN), the WHO, the United Nations Children's Fund (UNICEF) and government leaders in 2010 planned a series of low-cost and high-impact interventions to improve neonatal and infant health quality (World Health Organization, 2015a). The lack of public health structure in developing countries, however, still causes problems, reducing the viability of the proposal. Congenital abnormalities and neglected diseases are somewhat comparable, as both problems do not receive the necessary attention and prevail in developing regions. In Brazil, income transfers helped millions of people out of extreme poverty, but are still far from solving public health problems in the country. An intervention is needed, especially in the peripheral regions, with the application of preventive policies (Di Renzo *et al.*, 2015). These policies may differ according to the countries’ characteristics, but they should by all means emphasize health education of professionals, and the public, investment in primary reproductive healthcare, pregnancy planning, basic sanitation, and reliable registries with epidemiological data on congenital anomalies.

## Supplementary material

The following online material is available for this article:

Table S1 - Main teratogen categories and respective embryo-fetal effects during pregnancy.

Table S2 - Cases involving congenital anomalies from 2008 to 2013.

Table S3 - Fetal deaths involving congenital anomalies from 2008 to 2013.

Table S4 - Infant hospitalizations involving congenital anomalies from 2008 to 2013.

Table S5 - Number of deaths of infant hospitalized involving congenital anomalies from 2008 to 2013.

Table S6 - Average cost in US dollars of hospitalizations involving congenital anomalies from 2008 to 2013
