# Supplementary material for: Teratogens: a public health issue – a Brazilian overview
Source: Genet Mol Biol. 2017 May 22;40(2):387–97. doi: 10.1590/1678-4685-GMB-2016-0179 (PMC5488458; doi:10.1590/1678-4685-GMB-2016-0179)
Supplement: Supplementary file 1 [file 1415-4757-gmb-1678-4685-GMB-2016-0179-Suppl01.pdf]

Table S1 - Main teratogen categories and respective embryo-fetal effects during pregnancy.

Categories	Teratogen	Characteristic	Effects	Reference
**Category I: Drugs and substances**	Alcohol	The enzyme alcohol dehydrogenase (ADH) converts alcohol to acetaldehyde, which inhibits DNA synthesis, amino acid transport from placenta to the fetus, besides interfering in brain development. The susceptibility is related to the amount of ADH, which have variations in their expression due to genetic differences in ADH alleles.	Deficiency in intrauterine growth and postnatal growth, cognitive abnormalities, leading to a set of characteristics called fetal alcohol syndrome (FAS), characterized by: alterations in facial appearance (small palpebral fissures, large epicanthal folds, small head, small upper jaw, smooth philtrum, thin upper lip), decreased muscle tone, poor coordination, heart defects (ventricular and atrial septal defects), late reasoning, speech, movement and social skills development. FAS is the main cause of intellectual disability.	(Rostand *et al.*, 1990; Sampson *et al.*, 1997)
	Tobacco	Nicotine is a vasoconstrictor interfering in intrauterine growth due to decreased perfusion fetal tissues, which may lead to placental abruption. Carbon monoxide present in the smoke of the cigarette also crosses the placenta, producing an increase in carboxyhemoglobin in blood.	Oral clefts, low birth weight, intrauterine growth retardation, spontaneous abortion, premature birth.	(Werler 1997; Nicoletti *et al.*, 2014)
	Marijuana	It is extracted from the *cannabis sativa* plant. Liposoluble active compound 8, 9-tetrahydrocannabinol crosses the placenta easily, reaching the fetus. This compound binds to cannabinoid receptors, acting on the analgesia route, as well as the anxiolytic route and immunological system.	Intrauterine growth restriction, cognitive and neurobehavioral imbalance, respiratory and hormonal disorders.	(Kuczkowski 2004)

Categories	Teratogen	Characteristic	Effects	Reference
	LSD	LSD (lysergic acid diethylamide) produces a series of distortions in the functioning of the brain, changing the psychic, circulatory and thermal functions.	Brain damage, abnormalities in the lower jaw, modification of facial contours, defects in the limbs and eyes, joint problems and miscarriage.	(McGlothlin *et al.*, 1970)
	Cocaine	Cocaine has vasoconstrictor activity, which may result in an interruption of blood flow to the fetus.	Placental abruption, intrauterine growth retardation, limb defects, vascular disorders, prematurity, respiratory problems, ileal atresia, defects in brain growth and central nervous system (CNS), neurobehavioral disorders.	(Rizk *et al.*, 1996; Behnke *et al.*, 2001)
	Thalidomide	(S) enantiomer of thalidomide is an angiogenesis inhibitor, affecting the following pathway: growth factor I (IGF-I), and fibroblast growth factor 2 (FGF-2). Stimulation of integrin subunit B3 genes transcription, which are responsible for stimulation of angiogenesis in the developing limb buds, which promotes the growth from the root	Phocomelia of upper and lower limbs, pre-axial polydactyly, trifalangeal thumb, facial hemangiomas, esophageal and duodenal atresia, cardiac defects, renal agenesis, urinary tract anomalies, genital defects, dental anomalies, ear anomalies, facial palsy, ophthalmoplegia, anophthalmia and microphthalmia.	(Stephens *et al.*, 2000; Kim and Scialli 2011)
	Antagonists of folic acid and folic acid deficiency	Folic acid has an important role in nucleic acid synthesis. It acts as a coenzyme in various cellular reactions. It is included in cellular division process, due to the role in biosynthesis of purines and pyrimidines, in the transfer of carbon in the metabolism of amino acids and nucleic acids. A deficiency of folic acid or the use of antagonistic drugs can impair cell growth and replication of the fetus	Neural tube defects, heart defects (ventricular septum), cleft lip, cleft palate and anencephaly.	(Scholl and Johnson 2000; Hernández-Díaz *et al.*, 2001)
	Sedatives	Especially drugs containing benzodiazepine, which	Cleft lip, cleft palate, congenital heart disease, intrauterine growth	(Eger 1991; Leppée *et al.*,

Categories	Teratogen	Characteristic	Effects	Reference
		acts selectively on polysynaptic pathways of the CNS. They have an anti-anxiety and calming effect.	restriction, as well as changes in facial appearance, similar to FAS.	2010)
	Isotretinoin	It is a retinoic acid, which acts in the induction and control of epithelial differentiation, in mucus-secreting tissue or keratinizing, in the production of prostaglandin E2, of collagen. Also acts in controlling the proliferation of certain skin microorganisms.	Cleft palate, webbed neck, microphthalmia and absent external ears, cardiovascular defects (aortic arch interruption, defect in the atrial and ventricular septum), hydrocephalus, absence of cerebellar vermis, defects in cranial nerves.	(Hansen and Pearl 1985)
	Caffeine	Caffeine is a xanthine alkaloid, which readily crosses the human placenta, entering the fetal circulation during its development. Drug effect occurs on the CNS stimulation, such as increased motor activity, catecholamine release, adrenaline and noradrenaline release, serotonin turnover in selected areas, inhibition of phosphodiesterase activity.	CNS defects, orofacial clefts, structural skeletal defects, cardiovascular malformations, adactyly and absence of thumbs.	(Al-Hachim 1989; Collier *et al.,* 2009)
	Ergotamine	It is a natural alkaloid, which acts on smooth muscle contraction, producing fetal vasoconstriction.	Neural tube defects, cardiovascular malformation, polydactyly, intrauterine growth retardation and jejunal atresia.	(Czeizel 1989)
	Phenytoin<br><br>(Hydantoin)	It is an antiepileptic drug that decreases the neuronal excitation. It stabilizes the neuronal membrane by inhibiting the sodium channel, interfering in the psychomotor performance.	Delayed mental development, craniofacial dysmorphism, hypoplasia of the distal phalanges, cardiac, skeletal and eye defects, imbalance in the immune system. Can cause a number of disruptions, known as the fetal hydantoin syndrome.	(Hansen and Billings 1985; Singh and Shah 1989; Hill *et al.,* 2010)

Categories	Teratogen	Characteristic	Effects	Reference
	Dimethadione and trimethadione	Anticonvulsants oxazolidinones that have action in the thalamus region by repetitive stimulation, acting on Ca^{2+} currents.	Spontaneous abortions, microphthalmia, anophthalmia, microcephaly, celosomia, absence of toe, developmental delay, facial alterations (brachycephaly, V-shaped eyebrows, broad nasal bridge, cleft palate, mal-positioned ears), cardiovascular defects (globular heart), renal malformations, ventral hernia, hypospadias and intellectual disabilities.	(Rifkind 1974)
	Warfarin	It is an anticoagulant, which readily crosses the placenta. It has an action in the coagulation cascade by reducing hepatic synthesis of factors II, VII, IX and X, besides the inhibition of vitamin K formation.	Spontaneous abortions; can cause the fetal warfarin syndrome (skeletal abnormalities, nasal hypoplasia, narrow nasal bridge, scoliosis, spinal calcifications, femur and heel bone calcifications, low birth weight, and developmental disabilities.	(Baillie *et al.,* 1980; Starling *et al.,* 2012)
	Angiotensin-converting enzyme (ACE) inhibitors	It is an antihypertensive that has an action in ACE inhibition, which is an enzyme responsible for converting angiotensin I to angiotensin II, acting on the renin angiotensin aldosterone system.	Small formation of amniotic fluid (oligohydramnios), spontaneous abortions, intrauterine and neonatal deaths, neonatal respiratory distress, central nervous system and limb defects, calcarial hypoplasia and renal disorders (intrauterine renal failure, renal tubular dysplasia).	(Barr 1994)
	Statins	Statins are used for reducing the serum levels of cholesterol by inhibiting 3-hydroxy-3-methylglutaryl-coenzyme A reductase (HMG - CoA) altering the kinetics of formation of cholesterol.	Spontaneous abortion, CNS defects, skeletal abnormalities, hypospadias, duodenal atresia, cleft lip and scars on the skin.	(Godfrey *et al.,* 2012)
	Misoprostol	It is a synthetic prostaglandin E1 (PGE1) used for stomach disorders, activating the	Neurological disorders (Moebius syndrome), malformations of limbs	(da Silva Dal Pizzol *et al.,* 2006; Allen and O'Brien

Categories	Teratogen	Characteristic	Effects	Reference
		production of protective mucus, as well as increasing blood flow. However, it can cause strong uterine contractions and is used as an illegal abortion method.	and miscarriage.	2009)
	Tetracycline	A broad-spectrum action antibiotic, bacteriostatic, which has an action in the bacterial protein synthesis inhibition by binding the 30S subunit of the bacterial ribosome. When used in late pregnancy, it causes antibiotic deposition in the calcification of teeth.	Modification in dental enamel with yellow-brown discoloration, and calcification of deciduous teeth	(Demers *et al.*, 1968)
	Lithium	Used in bipolar disorder treatment, crossing the placenta freely and affecting the vascular formation of the fetus, showing greater teratogenic potential in the first quarter of pregnancy.	Cardiac anomalies are more common, such as Epstein's anomaly, dextrocardia, coarctation of the aorta, as well as hypotonia, respiratory distress syndrome, cyanosis, muscle weakness and lethargy.	(Gentile 2012)
**Category II: Physical agents**	Radiation	Ionizing radiation can cause cellular death, gene mutation, and change in the mitosis pattern, which leads to serious damage to embryonic development.	Spontaneous abortion, intrauterine growth retardation, microcephaly, intellectual disabilities.	(Brent 1980; De Santis *et al.*, 2005)
**Category III: Environmental agents**	Lead	Accumulates in maternal bone tissue; It is released slowly, crossing the placenta between the 12th-14th weeks, accumulating in fetal tissue.	Spontaneous abortion, vertebral alterations and anal defects, cardiovascular defects, polydactyly, clubfoot, tracheoesophageal fistula, renal defects and abnormalities of limbs.	(Bellinger 2005)
	Mercury	Organic forms are more toxic than inorganic forms. Methylmercury (MeHg) readily crosses the placenta and the hematoencephalic barriers; In addition to being highly toxic, it is selective to the CNS, leading to inhibition of the neuronal cell, such as the	CNS defects, neurobehavioral disorders. The best known syndromic alteration is Minamata disease that includes sensory disturbances in the hands and feet, ocular and hearing disorders, weakness and paralysis	(Myers *et al.*, 2003; Bose-O'Reilly *et al.*, 2010; Sagiv *et al.*, 2014)

Categories	Teratogen	Characteristic	Effects	Reference
		division and migration. The contamination normally occurs by ingestion of poisoned food, such as fish.	of limbs.	
	Toluene	Toluene (methylbenzene) is a liposoluble aromatic hydrocarbon, which is used as a solvent, capable of easily crossing placental barrier, causing metabolic acidosis and hypoxia, which will lead to fetal hypoperfusion and ischemia	Prematurity, failure to thrive, microcephaly, anencephaly, developmental delay, renal disorders and craniofacial anomalies.	(Donald *et al.*, 1991; Wilkins-Haug 1997)
	Polychlorinated and polybrominated byphenyls (PCBs)	(PCBs) are synthetic hydrocarbons. They are chemical pollutants that have lipophilic characteristics, and may cross the placenta, reaching the fetus directly or be transferred via breast milk, accumulating mainly in the skin.	Hyperpigmentation and facial acne, nail alterations, and behavioral deficits intellectual disabilities.	(Jacobson and Jacobson 1997; Cohn *et al.*, 2011)
	Chromium	Chromium is used for biological applications and industrial processes. This chemical agent may reach the circulatory system and cross the placental barrier, reaching the fetus and transferring chromium from the mother to the bones of the developing fetus	Skeletal abnormalities, sub-dermal hemorrhagic patches and abortion	(Kanojia *et al.*, 1996)
**Category IV: Maternal infections**	Varicella	Caused by the *varicella-zoster* virus (VZV), which is able to cross the placenta and infect the fetus.	Skin lesions such as scars are more common, miscarriage, premature birth, muscular and skeletal malformations, defects in the CNS, calcifications, blindness, growth retardation and intellectual disabilities.	(Sauerbrei 2010)
	Mumps	Caused by the *paramyxovirus* virus RNA, which can reach the fetus in cases of acute maternal viremia. Mal-formative effects during	The effects observed are postnatal, causing inflammation of the parotid, submaxillary and sublingual glands.	(Ornoy and Tenenbaum 2006; Lozo *et al.*, 2012)

Categories	Teratogen	Characteristic	Effects	Reference
		pregnancy are unknown.		
	Influenza	Caused by influenza A, B and C, typically occurring during the winter, associated with periods of fever and secondary bacterial infections of the respiratory system. The influenza virus infection appears to have no significant effects in the fetal development.	The effects observed are postnatal periods which include fever and respiratory problems.	(Acs *et al.*, 2005)
	Cytomegalovirus	Caused by viruses of the family *Herpesviridae*, reaching the fetus in phases of acute viremia in the different embryonic stages.	Microcephaly, intellectual disabilities, unilateral or bilateral deafness, neuromuscular diseases, chorioretinitis, hepatosplenomegaly, cerebral calcifications, and cortical / subcortical abnormalities.	(Pass *et al.*, 1980; Pascual-Castroviejo *et al.*, 2012)
	Parvovirosis	Caused by the human Parvovirus B-19. It is able to cross the placenta and infect the liver of the fetus, which is the main hematopoietic source of the embryo. This virus has affinity for dividing cells, in particular the erythropoietic tissues.	Spontaneous abortion, fetal hydrops, fetal anemia, myocarditis, liver failure, CNS defects, craniofacial and eye abnormalities.	(Ergaz and Ornoy 2006)
	Syphilis	Caused by the *Treponema pallidum*, which is a spirochete capable of crossing the placental barrier and infecting the fetus near 14th week of gestation. Placental infection and reduced blood flow to the fetus are the causes of fetal death.	Spontaneous abortion, prematurity, low birth weight, hepatosplenomegaly, hematological disorders.	(Genc and Ledger 2000; De Santis *et al.*, 2012)
	Toxoplasmosis	Caused by the protozoan *Toxoplasma gondii*, the most common infection in e pregnancy. Transmission in adults occurs through the consumption of undercooked meat or contact with the feces of infected cats. The parasite crosses from the placenta	Seizures, intellectual disability, cerebral palsy, deafness and blindness.	(Yokota 1995; Rorman *et al.*, 2006)

Categories	Teratogen	Characteristic	Effects	Reference
		to infect the fetus.		
	Herpes	Herpes simplex virus (HSV) type 1/2 and Epstein - Barr virus (EBV) belong to human herpes virus. Genital HSV can cause intrauterine infection or during childbirth. Transplacental transmission of the EBV is rare, but can occur.	Effects caused by HSV include spontaneous abortion, skin manifestations, chorioretinitis, microphthalmia and neurological damage. EBV affects the heart, liver and eyes.	(Avgil and Ornoy 2006; Malm and Forsgren 2009)
	Genitourinary infections	The most common infections are bacterial vaginosis (*Gardnerella vaginalis, Bacteroides spp, Mycoplasma hominis, Mobiluncus* spp.) and candidiasis (*Candida albicans*); they may infect the fetus during childbirth.	Skin infections, dermatological alterations and ophthalmological problems.	(Hay 2005)
	*Zika virus	Zika virus (ZIKV) is a flavivirus of the same family as yellow fever, dengue, West Nile, and Japanese encephalitis viruses. The transmission occurs through the bite of the *Aedes spp.* mosquitoes, including *Ae. africanus, Ae. luteocephalus, Ae. hensilli,, Ae. Aegypti,* well as with potential sexual transmission. It leads to symptoms such as rash, arthralgia, and conjunctivitis. It is suspected to cause microcephaly in babies born from women that contracted the virus during pregnancy.	Microcephaly, neurological and ophthalmic anomalies	(Hayes 2009; Campos *et al.*, 2015; Musso *et al.*, 2015; Atkinson *et al.*, 2016; D’Ortenzio *et al.*, 2016; Freitas *et al.*, 2016; Mlakar *et al.*, 2016; Rasmussen *et al.*, 2016; Schuler-Faccini *et al.*, 2016; Ventura *et al.*, 2016)
**Category V: Maternal conditions**	Obesity	Excessive weight gain during pregnancy may impair intrauterine life, as well as maternal life, leading to serious complications such as preeclampsia and gestational diabetes.	Macrosomia and cardiovascular defects are more common. Obesity can also increase the risk of neural tube defects, orofacial clefts, hydrocephalus, anal atresia, hypospadias, renal abnormalities, omphalocele, and	(Stothard *et al.*, 2009; Blomberg and Källén 2010)

Categories	Teratogen	Characteristic	Effects	Reference
			diaphragmatic hernia.	
	Diabetes mellitus	Hyperglycemic state leads to an increase in the formation of glycated hemoglobin, in addition to increasing oxidative stress in embryos, inhibiting the expression of specific genes, such as Pax3, which encodes a transcription factor for neural tubes.	Spontaneous abortion, macrosomia, neural tube defects, CNS disorders, cardiovascular defects.	(Ray *et al.,* 2001; Loeken 2006)
	Hypothyroidism	Deficiency of thyroid stimulating hormone (TSH) can be caused by a problem in the development of the thyroid gland (dysgenesis) due to a mutation in the transcription factor of the thyroid 2 (TTF-2) or by a biosynthesis thyroid hormone disorder (dyshormonogenesis), having an effect on the neurological development of the fetus.	Choanal atresia, cleft palate, prolonged jaundice, difficulty feeding, lethargy, umbilical hernia, macroglossia constipation, blotchy skin, hypotonia.	(Bamforth *et al.,* 1986; Källén and Wikner 2014)
	Hyperthyroidism	Caused mainly by autoimmune diseases, such as Grave's disease, or in combination with other maternal biochemical disturbances. Antibodies and antithyroid medication given to the mother can cross the placenta and affect the fetal thyroid gland	Malformation of the ear lobes, omphalocele, imperforate anus, anencephaly, cleft lip, growth retardation, accelerated bone maturation, goiter and policactilia.	(Momotani *et al.,* 1984; Alamdari *et al.,* 2013)
	Hypoparathyroidism	The parathyroid dysfunction is caused by parathyroid hormone deficiency, which leads to hypocalcemia and hyperphosphatemia	Prematurity, bone demineralization, craniofacial uncovered, microcephaly, deep-set eyes, thin lips, micrognathia, flattened nasal bridge, anomalies in the outer ear, hand and feet small, micropenis and intellectual disabilities.	(Sanjad *et al.,* 1991)
	Iodine deficiency	During pregnancy, iodine intake should be increased by almost 50%. Iodine	Spontaneous abortion, stillbirth, genital abnormalities, hearing	(Hetzel and Mano 1989; Zimmermann

Categories	Teratogen	Characteristic	Effects	Reference
		deficiency during pregnancy can cause fetal hypothyroidism and impair neurological development of the fetus	disorders, brain function impaired, the most common being cretinism, which is an intellectual impairment, as well as the strabismus.	2009; Zimmermann 2012)
	Phenylketonuria	Characterized by decreased activity of the enzyme phenylalanine hydroxylase, which is responsible for transforming phenylalanine to tyrosine, which leads to accumulation of phenylalanine in the fetus	Intrauterine growth retardation, microcephaly, cardiovascular defects, intellectual disabilities.	(Levy and Ghavami 1996; Matalon *et al.*, 2003)
	Hypoglycemia	Stages of hypoglycemia during pregnancy stop the power supply to the fetus, as well as induced hypoxia.	Spontaneous abortion, intrauterine growth retardation.	(Zamudio *et al.*, 2010)
	Hyperthermia	Maternal body temperature above 39 ° C can lead to cell death or delay of the proliferation of neuroblasts. It can also lead to fetal vascular disruption.	Anencephaly, microphthalmia, arthrogryposis, abdominal wall defects, abnormalities of the distal limbs, midface hypoplasia and intellectual disabilities.	(Isaacs and Gericke 1990; Graham *et al.*, 1998; Edwards 2006)

*Zika virus was confirmed to cause an outbreak of microcephaly in newborns of women that contracted the virus during pregnancy (Rasmussen *et al.*, 2016). Here we present this virus as a new teratogenic agent. Shepard's criteria have already been contemplated (Rasmussen *et al.*, 2016), more epidemiological studies are being performed to establish the teratogenic potential and spectrum of malformations.

References

Acs N, Bánhidy F, Puhó E and Czeizel AE (2005) Maternal influenza during pregnancy and risk of congenital abnormalities in offspring. Birth Defects Res A Clin Mol Teratol 73:989-996.

Alamdari S, Azizi F, Delshad H, Sarvghadi F, Amouzegar A and Mehran L (2013) Management of hyperthyroidism in pregnancy: Comparison of recommendations of american thyroid association and endocrine society. J Thyroid Res 2013:878467.

Al-Hachim GM (1989) Teratogenicity of caffeine; a review. Eur J Obstet Gynecol Reprod Biol 31:237-247.

Allen R and O'Brien BM (2009) Uses of misoprostol in obstetrics and gynecology. Rev Obstet Gynecol 2:159-168.

Atkinson B, Hearn P, Afrough B, Lumley S, Carter D, Aarons EJ, Simpson AJ, Brooks TJ and Hewson R (2016) Detection of Zika Virus in semen. Emerg Infect Dis 22:160107.

Avgil M and Ornoy A (2006) Herpes simplex virus and Epstein-Barr virus infections in pregnancy: Consequences of neonatal or intrauterine infection. Reprod Toxicol 21:436-445.

Baillie M, Allen ED and Elkington AR (1980) The congenital warfarin syndrome: A case report. Br J Ophthalmol 64:633-635.

Bamforth JS, Hughes I, Lazarus J and John R (1986) Congenital anomalies associated with hypothyroidism. Arch Dis Child 61:608-609.

Barr M (1994) Teratogen update: Angiotensin-converting enzyme inhibitors. Teratology 50:399-409.

Behnke M, Eyler FD, Garvan CW and Wobie K (2001) The search for congenital malformations in newborns with fetal cocaine exposure. Pediatrics 107:e74.

Bellinger DC (2005) Teratogen update: Lead and pregnancy. Birth Defects Res A Clin Mol Teratol 73:409-420.

Blomberg MI and Källén B (2010) Maternal obesity and morbid obesity: The risk for birth defects in the offspring. Birth Defects Res A Clin Mol Teratol 88:35-40.

Bose-O'Reilly S, McCarty KM, Steckling N and Lettmeier B (2010) Mercury exposure and vhildren's health. Curr Probl Pediatr Adolesc Health Care 40:186-215.

Brent RL (1980) Radiation teratogenesis. Teratology 21:281-98.

Campos GS, Bandeira AC and Sardi SI (2015) Zika virus outbreak, Bahia, Brazil. Emerg Infect Dis 21:1885-1886

Cohn B, Cirillo P, Sholtz R, Ferrara A, Park J and Schwingl P (2011) Polychlorinated biphenyl (PCB) exposure in mothers and time to pregnancy in daughters. Reprod Toxicol 31:290-296.

Collier SA, Browne ML, Rasmussen SA and Honein MA (2009) Maternal caffeine intake during pregnancy and orofacial clefts. Birth Defects Res A Clin Mol Teratol 85:842-849.

Czeizel A (1989) Teratogenicity of ergotamine. J Med Genet 26:69-70.

D'Ortenzio E, Matheron S, Lamballerie X de, Hubert B, Piorkowski G, Maquart M, Descamps D, Damond F, Yazdanpanah Y and Leparc-Goffart I (2016) Evidence of sexual transmission of Zika virus. N Engl J Med 374:2195-2198.

da Silva Dal Pizzol T, Knop FP and Mengue SS (2006) Prenatal exposure to misoprostol and congenital anomalies: Systematic review and meta-analysis. Reprod Toxicol 22:666-671.

De Santis M, De Luca C, Mappa I, Spagnuolo T, Licameli A, Straface G and Scambia G (2012) Syphilis Infection during pregnancy: Fetal risks and clinical management. Infect Dis Obstet Gynecol 2012:430585.

De Santis M, Di Gianantonio E, Straface G, Cavaliere AF, Caruso A, Schiavon F, Berletti R and Clementi M (2005) Ionizing radiations in pregnancy and teratogenesis: A review of literature. Reprod Toxicol 20:323-329.

Demers P, Fraser D, Goldbloom R, Haworth JC, LaRochelle J, MacLean R and Murray T (1968) Effects of tetracyclines on skeletal growth and dentition: A report by the Nutrition Committee of the Canadian Paediatric Society. Can Med Assoc J 99:849-854.

Donald JM, Hooper K and Hopenhayn-Rich C (1991) Reproductive and developmental toxicity of toluene: A review. Environ Health Perspect 94:237-244.

Edwards MJ (2006) Review: Hyperthermia and fever during pregnancy. Birth Defects Res A Clin Mol Teratol 76:507-516.

Eger EI (1991) Fetal injury and abortion associated with occupational exposure to inhaled anesthetics. AANA J 59:309-312.

Ergaz Z and Ornoy A (2006) Parvovirus B19 in pregnancy. Reprod Toxicol 21:421-435.

Freitas B de P, Dias JR de O, Prazeres J, Sacramento GA, Icksang KA, Maia M and Belfort R (2016) Ocular findings in infants with microcephaly associated with presumed Zika virus congenital. JAMA Ophthalmol. 134:529-535.

Genç M and Ledger WJ (2000) Syphilis in pregnancy. Sex Transm Infect 76:73-79.

Gentile S (2012) Lithium in pregnancy: The need to treat, the duty to ensure safety. Expert Opin Drug Saf 11:425-437.

Godfrey LM, Erramouspe J and Cleveland KW (2012) Teratogenic risk of statins in pregnancy. Ann Pharmacother 46:1419-1424.

Graham JM, Edwards MJ and Edwards MJ (1998) Teratogen update: Gestational effects of maternal hyperthermia due to febrile illnesses and resultant patterns of defects in humans. Teratology 58:209-221.

Hansen DK and Billings RE (1985) Phenytoin teratogenicity and effects on embryonic and maternal folate metabolism. Teratology 31:363-371.

Hansen GS and Pearl LA (1985) Isotretinoin teratogenicity. Acta Neuropathol 65:335-337.

Hay P (2005) Genito-urinary infections in pregnancy. Women's Heal Med 2:47-50.

Hayes EB (2009) Zika virus outside Africa. Emerg Infect Dis 15:1347-1350.

Hernández-Díaz S, Werler M, Walker A and AA. M (2001) Neural tube defects in relation to use of folic acid antagonists during pregnancy. Am J Epidemiol 153:961-968.

Hetzel BS and Mano MT (1989) A review of experimental studies of iodine deficiency during fetal development. J Nutr 119:145-151.

Hill DS, Wlodarczyk BJ, Palacios AM and Finnell RH (2010) Teratogenic effects of antiepileptic drugs. Expert Rev Neurother 10:943-959.

Isaacs H and Gericke G (1990) Concurrence of malignant hyperthermia and congenital abnormalities. Muscle Nerve 13:915-917.

Jacobson JL and Jacobson SW (1997) Teratogen update: Polychlorinated biphenyls. Teratology 55:338-347.

Källén B and Wikner BN (2014) Maternal hypothyroidism in early pregnancy and infant structural congenital malformations. J Thyroid Res 2014:160780.

Kanojia RK, Junaid M and Murthy RC (1996) Chromium induced teratogenicity in female rat. Toxicol Lett 89:207-213.

Kim JH and Scialli AR (2011) Thalidomide: The tragedy of birth defects and the effective treatment of disease. Toxicol Sci 122:1-6.

Kuczkowski KM (2004) Marijuana in pregnancy. Ann Acad Med Singapore 33:336-339.

Leppée M, Culig J, Eric M and Sijanovic S (2010) The effects of benzodiazepines in pregnancy. Acta Neurol Belg 110:163-167.

Levy HL and Ghavami M (1996) Maternal phenylketonuria: A metabolic teratogen. Teratology 53:176-184.

Loeken MR (2006) Advances in understanding the molecular causes of diabetes-induced birth defects. J Soc Gynecol Investig 13:2-10.

Lozo S, Ahmed A, Chapnick E, O'Keefe M and Minkoff H (2012) Presumed cases of mumps in pregnancy: Clinical and infection control implications. Infect Dis Obstet Gynecol 2012:345068.

Malm G and Forsgren M (2009) Neonatal herpes simplex virus infection. Arch Dis Child Fetal Neonatal 14:204-208.

Matalon KM, Acosta PB and Azen C (2003) Role of Nutrition in Pregnancy With Phenylketonuria and Birth Defects. Pediatrics 112:1534-1536.

McGlothlin WH, Sparkes RS and Arnold DO (1970) Effect of LSD on Human Pregnancy. JAMA 212:1483-1487.

Mlakar J, Korva M, Tul N, Popović M, Poljšak-Prijatelj M, Mraz J, Kolenc M, Resman RK, Vesnaver VT, Vodušek VF, *et al.*(2016) Zika Virus Associated with Microcephaly. N Engl J Med 374:951-958.

Momotani N, Ito K, Hamada N, Ban Y, Nishikawa Y and Mimura T (1984) Maternal hyperthyroidism and congenital malformation in the offspring. Clin Endocrinol (Oxf) 20:695-700.

Musso D, Roche C, Robin E, Nhan T, Teissier A and Cao-Lormeau VM (2015) Potential sexual transmission of zika virus. Emerg Infect Dis 21:359-361.

Myers GJ, Davidson PW, Cox C, Shamlaye CF, Palumbo D, Cernichiari E, Sloane-Reeves J, Wilding GE, Kost J, Huang LS, *et al.* (2003) Prenatal methylmercury exposure from ocean fish consumption in the Seychelles child development study. Lancet 361:1686-1692.

Nicoletti D, Appel LD, Neto PS, Guimarães GW and Zhang L (2014) Tabagismo materno na gestação e malformações congênitas em crianças: Uma revisão sistemática com meta-análise. Cad Saude Publica 30:1-40.

Ornoy A and Tenenbaum A (2006) Pregnancy outcome following infections by coxsackie, echo, measles, mumps, hepatitis, polio and encephalitis viruses. Reprod Toxicol 21:446-457.

Pascual-Castroviejo I, Pascual-Pascual SI, Velazquez-Fragua R and Viaño Lopez J (2012) Congenital cytomegalovirus infection and cortical/subcortical malformations. Neurologia 27:336-342.

Pass RF, Stagno S, Myers GJ, Alford CA, Myers J and Alford A (1980) Outcome of symptomatic congenital Cytomegalovirus infection: Results of long-term longitudinal follow-up. Pediatrics 66:758-762.

Rasmussen SA, Jamieson DJ, Honein MA and Petersen LR (2016) Zika virus and birth defects - Reviewing the evidence for causality. N Engl J Med 374:1981-1987.

Ray JG, O'Brien TE and Chan WS (2001) Preconception care and the risk of congenital anomalies in the offspring of women with diabetes mellitus: A meta-analysis. QJM 94:435-444.

Rifkind AB (1974) Teratogenic effects of: Trimethadione and Dimethadione in the chick embryo. Toxicol Appl Pharmacol 30:452-457.

Rizk B, Atterbury JL and Groome LJ (1996) Reproductive risks of cocaine. Hum Reprod Update 2:43-55.

Rorman E, Zamir C, Rilkis I and Bendavid H (2006) Congenital toxoplasmosis - Prenatal aspects of Toxoplasma gondii infection. Reprod Toxicol 21:458-472.

Rostand A, Kaminski M, Lelong N, Dehaene P, Delestret I, Klein-Bertrand C, Querleu D and Crepin G (1990) Alcohol use in pregnancy, craniofacial features, and fetal growth. J Epidemiol Community Health 44:302-306.

Sagiv SK, Thurston SW, Bellinger DC, Amarasiriwardena C and Korrick SA (2014) Prenatal exposure to mercury and fish consumption during pregnancy and ADHD-related behavior in children. Arch Pediatr Adolesc Med 166:1123-1131.

Sampson PD, Streissguth AP, Bookstein FL, Little RE, Clarren SK, Dehaene P, Hanson JW and Graham JM (1997) Incidence of fetal alcohol syndrome and prevalence of alcohol-related neurodevelopmental disorder. Teratology 56:317-326.

Sanjad SA, Sakati NA, Abu-Osba YK, Kaddoura R and Milner RD (1991) A new syndrome of congenital hypoparathyroidism, severe growth failure, and dysmorphic features. Arch Dis Child 66:193-196.

Sauerbrei A (2010) Review of varicella-zoster virus infections in pregnant women and neonates. Health (Irvine Calif) 2:143-152.

Scholl TO and Johnson WG (2000) Folic acid: Influence on the outcome of pregnancy. Am J Clin Nutr 71:1295S-303S.

Schuler-Faccini L, Ribeiro EM, Feitosa IML, Horovitz DDG, Cavalcanti DP, Pessoa A, Doriqui MJR, Neri JI, Neto JM de P, Wanderley HYC, *et al.* (2016) Possible association between Zika virus infection and microcephaly - Brazil, 2015. MMWR Morb Mortal Wkly Rep 65:59-62.

Singh M and Shah GL (1989) Teratogenic effects of phenytoin on chick embryos. Teratology 40:453-458.

Starling LD, Sinha A, Boyd D and Furck A (2012) Fetal warfarin syndrome. BMJ Case Rep 2012:691-695.

Stephens TD, Bunde CJ and Fillmore BJ (2000) Mechanism of action in thalidomide teratogenesis. Biochem Pharmacol 59:1489-1499.

Stothard KJ, Tennant PWG, Bell R and Rankin J (2009) Maternal overweight and obesity and the risk of congenital anomalies: A systematic review and meta-analysis. JAMA 301:636-650.

Ventura CV, Maia M, Ventura BV, Linden V Van Der, Araújo EB, Ramos RC, Rocha MAW, Carvalho MDCG, Belfort R and Ventura LO (2016) Ophthalmological findings in infants with microcephaly and presumable intra-uterus Zika virus infection. Arq Bras Oftalmol 79:1-3.

Werler MM (1997) Teratogen update: Smoking and reproductive outcomes. Teratology 55:382-388.

Wilkins-Haug L (1997) Teratogen update: Toluene. Teratology 55:145-151.

Yokota K (1995) Congenital anomalies induced by Toxoplasma infection. Congenit Anom (Kyoto) 35:151-168.

Zamudio S, Torricos T, Fik E, Oyala M, Echalar L, Pullockaran J, Tutino E, Martin B, Belliappa S, Balanza E, *et al.* (2010) Hypoglycemia and the origin of hypoxia-induced

reduction in human fetal growth. PLoS One 5:e8551.

Zimmermann MB (2009) Iodine deficiency in pregnancy and the effects of maternal iodine supplementation on the offspring: A review. Am J Clin Nutr 89:668-672.

Zimmermann MB (2012) The effects of iodine deficiency in pregnancy and infancy. Paediatr Perinat Epidemiol 26:108-117.
