# Supplementary material for: Teratogens: a public health issue – a Brazilian overview
Source: Genet Mol Biol. 2017 May 22;40(2):387–97. doi: 10.1590/1678-4685-GMB-2016-0179 (PMC5488458; doi:10.1590/1678-4685-GMB-2016-0179)
Supplement: Supplementary file 2 [file 1415-4757-gmb-1678-4685-GMB-2016-0179-Suppl02.pdf]

Table S2 - Cases involving congenital anomalies from 2008 to 2013.

**Birth defects**	**2008**	**2009**	**2010**	**2011**	**2012**	**2013**
Spina bifida	448	461	545	559	555	569
Other congenital malformations of the nervous system	1864	2000	1886	2053	1945	1855
Congenital malformations of the circulatory system	1083	1287	1377	1633	1881	2509
Cleft lip and cleft palate	1311	1423	1513	1593	1524	1587
Congenital absence, atresia, and stenosis of small intestine	40	41	47	45	47	47
Other congenital malformations of the digestive system	854	827	925	1042	923	954
Other malformations of the genitourinary system	1508	1673	1792	1870	1799	1835
Congenital abnormalities of the hip	86	103	90	116	113	139
Congenital abnormalities of the feet	2738	2937	2878	3031	2831	2802
Other congenital malformations of the musculoskeletal system	5376	5770	6288	6376	6262	6359
Other congenital malformations	2437	2663	2594	2922	2945	3020
Hemangioma and lymphangioma	107	107	88	101	104	108
Congenital syphilis	0	0	0	2		0
Dentofacial anomalies	0	0	0	1	1	0
Other specific tegumental affections in newborns		0	0	3	5	0
**Total**	17852	19292	20023	21347	20935	21784

Source: Ministério da Saúde - Sistema de Informações Sobre Nascidos Vivos (Sinasc)
