# Supplementary material for: Teratogens: a public health issue – a Brazilian overview
Source: Genet Mol Biol. 2017 May 22;40(2):387–97. doi: 10.1590/1678-4685-GMB-2016-0179 (PMC5488458; doi:10.1590/1678-4685-GMB-2016-0179)
Supplement: Supplementary file 3 [file 1415-4757-gmb-1678-4685-GMB-2016-0179-Suppl06.pdf]

Table S6 - Average cost in US dollars of hospitalizations involving congenital anomalies from 2008 to 2013

**Birth defects**	**2008**	**2009**	**2010**	**2011**	**2012**	**2013**
Spina bifida	504.07	522.13	673.53	682.16	656.29	713.25
Other congenital malformations of the nervous system	680.47	830.35	801.78	879.47	923.52	942.39
Congenital malformations of the circulatory system	1673.30	2122.50	2242.37	2628.47	2656.80	2724.60
Cleft lip and cleft palate	305.19	311.90	309.99	355.70	362.03	371.01
Congenital absence, atresia, and stenosis of small intestine	872.60	1732.54	2464.45	1007.11	1201.38	1627.58
Other congenital malformations of the digestive system	412.63	528.24	569.02	569.95	641.43	697.58
Other malformations of the genitourinary system	119.40	152.31	157.53	162.69	167.75	173.91
Congenital abnormalities of the hip	239.18	250.16	322.26	312.03	290.71	334.73
Congenital abnormalities of the feet	90.28	96.17	95.78	95.64	97.03	108.62
Other congenital malformations of the musculoskeletal system	218.79	253.40	285.49	310.54	343.94	354.62
Other congenital malformations	125.83	170.42	178.76	172.12	179.57	194.75
Congenital syphilis	136.82	177.32	185.36	230.13	239.75	192.15
Infectious and parasitic diseases congenital	245.66	346.46	388.91	376.93	411.76	366.99
Total	5624.21	7493.91	8675.23	7782.93	8171.97	8802.18

Source: Ministério da Saúde - Sistema de Informações Hospitalares do SUS (SIH/SUS)
***Currency rate as of March 15, 2016: $1.00 = R$ 3.75**.
