# Supplementary material for: Teratogens: a public health issue – a Brazilian overview
Source: Genet Mol Biol. 2017 May 22;40(2):387–97. doi: 10.1590/1678-4685-GMB-2016-0179 (PMC5488458; doi:10.1590/1678-4685-GMB-2016-0179)
Supplement: Table S3 [file 1415-4757-gmb-1678-4685-GMB-2016-0179-Suppl03.pdf]

Table S3 - Fetal deaths involving congenital anomalies from 2008 to 2013.

**Birth defects**	**2008**	**2009**	**2010**	**2011**	**2012**	**2013**
Hydrocephalus and congenital spina bifida	85	89	73	84	99	58
Other congenital malformations of the nervous system	399	362	401	367	388	398
Congenital malformations of heart	128	133	145	131	159	169
Other congenital malformations of the circulatory system	13	14	18	21	22	23
Other congenital malformations	830	882	898	880	960	949
**Total**	1455	1480	1535	1483	1628	1597

Source: Ministério da Saúde - Sistema de Informações sobre Mortalidade (SIM)
