# Supplementary material for: Teratogens: a public health issue – a Brazilian overview
Source: Genet Mol Biol. 2017 May 22;40(2):387–97. doi: 10.1590/1678-4685-GMB-2016-0179 (PMC5488458; doi:10.1590/1678-4685-GMB-2016-0179)
Supplement: Table S4 [file 1415-4757-gmb-1678-4685-GMB-2016-0179-Suppl04.pdf]

Table S4 - Infant hospitalizations involving congenital anomalies from 2008 to 2013.

**Birth defects**	**2008**	**2009**	**2010**	**2011**	**2012**	**2013**
Spina bifida	856	914	751	1001	1034	932
Other congenital malformations of the nervous system	2411	2677	2599	2828	2650	2858
Congenital malformations of the circulatory system	15263	16436	15987	16188	15733	15457
Cleft lip and cleft palate	7943	7882	7573	8202	7937	8665
Congenital absence, atresia, and stenosis of small intestine	26	18	30	25	33	35
Other congenital malformations of the digestive system	3457	3903	3969	4149	4008	4296
Other malformations of the genitourinary system	10472	10659	10680	10153	9299	8984
Congenital abnormalities of the hip	2148	1511	1270	1336	1321	1364
Congenital abnormalities of the feet	5456	5380	5486	5273	5175	5458
Other congenital malformations of the musculoskeletal system	5387	5807	5857	6064	5884	6374
Other congenital malformations	16105	15889	16030	15978	15571	14947
Congenital syphilis	3570	3715	3938	4812	5853	7443
Infectious and parasitic diseases congenital	5379	6684	7543	8347	8162	9224
Total	78473	81475	81713	84356	82660	86037

Source: Ministério da Saúde - Sistema de Informações Hospitalares do SUS (SIH/SUS)
