# Supplementary material for: Teratogens: a public health issue – a Brazilian overview
Source: Genet Mol Biol. 2017 May 22;40(2):387–97. doi: 10.1590/1678-4685-GMB-2016-0179 (PMC5488458; doi:10.1590/1678-4685-GMB-2016-0179)
Supplement: Table S5 [file 1415-4757-gmb-1678-4685-GMB-2016-0179-Suppl05.pdf]

Table S5 - Number of deaths of infant hospitalized involving congenital anomalies from 2008 to 2013.

**Birth defects**	**2008**	**2009**	**2010**	**2011**	**2012**	**2013**
Spina bifida	24	25	29	27	23	13
Other congenital malformations of the nervous system	216	228	237	252	267	257
Congenital malformations of the circulatory system	1214	1342	1258	1202	1189	1086
Cleft lip and cleft palate	1	4	1	5	4	5
Congenital absence, atresia, and stenosis of small intestine	4	2	2	5	5	0
Other congenital malformations of the digestive system	121	143	159	143	145	183
Other malformations of the genitourinary system	30	62	49	54	51	54
Congenital abnormalities of the hip	12	11	6	1	3	2
Congenital abnormalities of the feet	5	5	5	2	2	1
Other congenital malformations of the musculoskeletal system	68	78	96	102	124	126
Other congenital malformations	117	103	116	124	136	141
Congenital syphilis	28	29	15	23	33	39
Infectious and parasitic diseases congenital	159	233	213	250	262	259
Total	1999	2265	2186	2190	2244	2166

Source: Ministério da Saúde - Sistema de Informações Hospitalares do SUS (SIH/SUS)
